# Whole-genome scan reveals significant non-additive effects for sire conception rate in Holstein cattle

**DOI:** 10.1186/s12863-018-0600-4

**Published:** 2018-02-27

**Authors:** Paula Nicolini, Rocío Amorín, Yi Han, Francisco Peñagaricano

**Affiliations:** 1 Department of Animal Sciences, University of Florida, 2250 Shealy Drive, Gainesville, FL 32611 USA; 2 Polo de Desarrollo Universitario, Universidad de la República, Tacuarembó, Uruguay; 3 University of Florida Genetics Institute, University of Florida, Gainesville, FL 32610 USA

**Keywords:** Bovine sperm, Dairy bull fertility, Dominance effect, Genome-wide study

## Abstract

**Background:**

Service sire has a considerable impact on reproductive success in dairy cattle. Most gene mapping studies for bull fertility have focused on additive effects, while non-additive effects have been largely ignored. The main goal of this study was to assess the relevance of non-additive effects on Sire Conception Rate (SCR) in Holstein dairy cattle. The analysis included 7.5 k Holstein bulls with both SCR records and 57.8 k single nucleotide polymorphism (SNP) markers spanning the entire genome.

**Results:**

The importance of non-additive effects was evaluated using an efficient two-step mixed model-based approach. Four genomic regions located on chromosomes BTA8, BTA9, BTA13 and BTA17 showed marked dominance and/or recessive effects. Most of these regions harbor genes, such as *ADAM28*, *DNAJA1*, *TBC1D20*, *SPO11*, *PIWIL3* and *TMEM119,* that are directly implicated in testis development, male germ line maintenance, and sperm maturation.

**Conclusions:**

This study provides further evidence for the relevance of non-additive effects in fitness-related traits, such as male fertility. In addition, these findings may point out new strategies for improving service sire fertility in dairy cattle via marker-assisted selection.

## Background

Reproduction efficiency is arguably a very important economic trait in dairy cattle. Reproductive inefficiency results in increased calving intervals, increased involuntary culling rates, decreased milk production, and delayed genetic progress, among other problems. All this causes significant economic losses [1]. It is clear hence that improving reproductive performance is of paramount importance for the dairy industry worldwide. Most reproductive studies have focused on cow fertility, while bull fertility has been largely ignored. However, it is known that service sire directly influences not only the fertilization process but also the viability of the preimplantation embryo [2]. Indeed, some studies have reported that a significant percentage of reproductive failure is attributable to bull subfertility [3–5]. Therefore, the fertility of service sires should not be overlooked in breeding schemes aimed at improving the reproductive performance of dairy cattle.

There is growing evidence that genetic factors explain part of the differences in fertility among dairy sires. Indeed, some semen production and quality traits, such as sperm concentration, sperm motility, and percentage of abnormal sperm, have moderate to high heritability [6]. In addition, several transcriptomic and proteomic studies have revealed numerous differences between the spermatozoa of high- and low-fertility bulls [7–9]. Moreover, multiple alternative approaches have been applied to identify genetic polymorphisms associated with bull fertility. In fact, both candidate gene studies [10–12] and whole-genome scans [13–17] have identified significant genomic regions and individual genes associated with service sire fertility. For instance, using a comparative genomic approach, two conserved spermatogenesis genes, *MAP1B* and *PPP1R11*, were implicated in male fertility in Holsteins [12]. A recent study has identified novel genetic variants in both β-defensin and *FOXJ3* genes related to bull sperm function [16]. Our group have identified regions in BTA21 and BTA25 strongly associated with service sire fertility in US Holstein bulls [17]. These regions harbor genes with known roles in spermatogenesis and fertilization. Given that these association studies only detect the most significant markers, and hence, the vast majority of the variants remain hidden, some studies have explored bull fertility using a pathway-based approach, a methodology based on testing the association of sets of functionally related genes. These pathway-based studies have revealed biological processes and molecular mechanisms that may explain part of the differences in male fertility in cattle [17, 18]. Overall, current evidence suggests that bull fertility is influenced by genetic factors, and hence it could be improved by genetic means.

It is important to remark that association studies primarily focus on the identification of genetic variants with additive effects. On the other hand, non-additive genetic effects receive in general much less attention. Indeed, it remains controversial the relative importance of non-additive effects on complex traits [19, 20]. It is believed that non-additive effects are important for traits closely related to fitness, including reproduction. As such, the main objective of this study was to investigate the importance of non-additive effects on service sire in Holstein cattle. Sire Conception Rate (SCR) was utilized as a measured of sire fertility. The relevance of non-additive effects, namely dominance, recessive and overdominance effects on SCR was investigated on a genome-wide scale using an efficient two-step mixed model-based approach.

## Methods

### Phenotypic and genotypic data

Since August 2008, the US dairy industry has access to a national phenotypic evaluation of service sire fertility called Sire Conception Rate (SCR). This bull fertility evaluation is exclusively based on cow field data. The current model considers not only factors closely related to the bull under evaluation (e.g., age and AI stud), but also different factors (nuisance variables) associated with the cow that receives the unit of semen, such as cow age, parity, and milk yield [21, 22]. The trait SCR is defined as the expected difference in conception rate of a given bull compared to the mean of all other evaluated bulls.

A total of 7458 Holstein bulls with SCR data were used in this study. Sire conception rate values ranged from − 16.6% to + 14.1%. These SCR records were obtained from 27 consecutive evaluations provided to the US dairy industry between August 2008 and August 2017. These bull fertility evaluations are available at the Council of Dairy Cattle Breeding (CDCB) website (https://www.uscdcb.com/). For bulls with multiple fertility evaluations, the most reliable SCR record (i.e. the SCR record with most breedings) was used in the analyses.

Genome-wide single nucleotide polymorphism (SNP) data for the 7458 Holstein bulls were provided by the Cooperative Dairy DNA Repository (CDDR). SNP information (name, chromosome and position) was based on the bovine genome reference UMD3.1. Markers that mapped to the sex chromosomes, or were monomorphic, or had minor allele frequency less than 1% were removed from the dataset. After data editing, a total of 57,838 SNP markers were retained for subsequent analysis.

### Modelling additive and non-additive effects

**Fig. 1:**
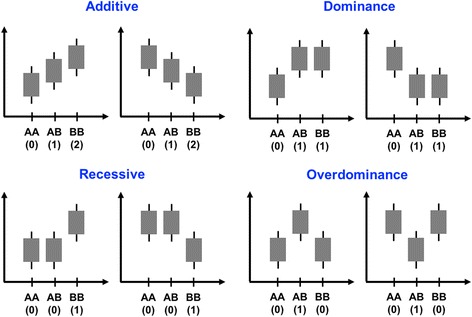
Overview of alternative genetic effects, namely additive, dominance, recessive, and overdominance, considering a single SNP locus with two alleles **A** and **B**. Note that in each scenario, the SNP genotype can be coded using a single numeric variable. Adapted from Tsepilow and coworkers [26]

Figure 1 provides an overview of alternative genetic effects, namely additive, dominance, recessive, and overdominance, considering a single SNP locus with two alleles A and B. In each scenario, the SNP genotype can be represented using a single numeric variable, and hence, these four effects can be easily tested on a genome-wide scale using alternative single-marker regressions. For testing the additive effect, i.e., the existence of a linear relationship between the number of allele copies and the phenotype, the three possible SNP genotypes can be coded as 0 for AA, 1 for AB and 2 for BB. In the case of the dominance effect, the SNP genotypes can be represented as 0 for AA, 1 for AB and 1 for BB. Note that in this scenario, we are testing if a single copy of the reference allele (B allele) has the same effect on the phenotype as two copies. Contrary, for the recessive effect, we want to test if zero copies of the reference allele have the same effect as a single copy, and hence, the SNP genotypes should be coded as 0 for AA, 0 for AB, and 1 for BB. Finally, for evaluating an overdominance effect, i.e., when the two homozygous genotypes have the same effect on the phenotype, but the heterozygous genotype either increases or decreases the trait, the SNP locus should be coded as 0 for both AA and BB, and 1 for AB.

### Two-step mixed model-based analysis

The association between phenotypes and SNP genotypes using related individuals can be implemented within the framework of the classical animal model, **y** = **Xβ** + **Zu** + **e**, where y is the vector of SCR records, **β** is the vector of fixed effects included in the model, u is the vector of random animal effects, and e is the vector of random residual effects. The matrices **X** and **Z** are the incidence matrices relating phenotypic records to fixed and animal effects, respectively. The random effects are assumed multivariate normal with $\mathbf{u}\sim N\left(0,\mathbf{K}{\boldsymbol{\upsigma}}_u^2\right)$ and $\mathbf{e}\sim N\left(0,\mathbf{R}{\boldsymbol{\upsigma}}_e^2\right)$, where ${\sigma}_u^2$ and ${\sigma}_e^2$ are the animal additive genetic and residual variances respectively; **K** is a kinship matrix that can be calculated using either pedigree or genotypic information, and **R** is typically an identity or a diagonal matrix.

For the whole-genome scan, the model can be rewritten as, **y** = **Xβ** + *X*_*SNP*_*β*_*SNP*_ + **Zu** + **e**, where *X*_*SNP*_ is the design matrix for the SNP under study and *β*_*SNP*_ is the regression coefficient or SNP effect. In this context, the significant effect of the SNP marker can be tested using a standard Wald statistic computed from the ratio of the estimate of *β*_*SNP*_ and its standard error. However, in our case, the application of this test across the whole genome is computationally demanding, and hence, a two-step mixed model-based approach was implemented [23, 24].

At the first step, the following model, **y** = **Xβ** + **Zu** + **e**, without including SNP was fitted. The random effects were assumed multivariate normal with $\mathbf{u}\sim N\left(0,\mathbf{G}{\sigma}_u^2\right)$ and $\mathbf{e}\sim N\left(0,\mathbf{I}{\sigma}_e^2\right)$, where **G** is the genomic relationship matrix and **I** an identity matrix. Note that for computational reasons, the reliabilities of the SCR values were not considered in the analysis, and hence, the **R** matrix was reduced to an identity matrix. The variance-covariance matrix was then estimated as ${\mathbf{V}}_{\mathbf{0}}=\mathbf{ZG}{\mathbf{Z}}^{\prime }{\sigma}_u^2+\mathbf{I}{\sigma}_e^2$.

At the second step, the following model was fitted for every SNP, **y** = **Xβ** + *X*_*SNP*_*β*_*SNP*_ + **ϵ**, assuming $\mathbf{e}\sim N\left(0,{\mathbf{V}}_o{\upsigma}_{\mathrm{e}}^2\right)$. The significance of the SNP effect under consideration was evaluated using the following test statistic,

$$ \mathbf{z}=\frac{{\mathbf{X}}_{\mathbf{SNP}}^{\prime }{\mathbf{V}}_{\mathbf{o}}^{-\mathbf{1}}\left(\mathbf{y}-\mathbf{X}\widehat{\boldsymbol{\upbeta}}\right)}{\sqrt{{\mathbf{X}}_{\mathbf{SNP}}^{\prime }{\mathbf{V}}_{\mathbf{o}}^{-\mathbf{1}}{\mathbf{X}}_{\mathbf{SNP}}}} $$

which approximates the Wald test, and hence, is asymptotically standard normal. These analyses were performed using the *R* package *MixABEL* [25].

The genomic control procedure was applied to correct for a possible inflation of the test statistics using the function VIFGC implemented in the R package *GenABEL* [26]. Bonferroni correction (0.01/57,838 = 1.7*e*^−07^) was used to control for multiple testing.

## Results and discussion

Improving reproductive performance remains a major goal in dairy cattle. Service sire has been recognized as an important factor affecting herd fertility. Selective breeding is one strategy for improving bull fertility. Indeed, recent studies have identified and evaluated potential fertility-related biomarkers that could be used to select high fertility bulls [16, 17, 27]. It should be recognized that gene mapping studies have primarily focused on genetic variants with additive effects, whereas the role of non-additive effects has been largely overlooked. However, the theory suggests that non-additive effects could be important for fitness-related traits, such as fertility. As such, this study was specially conducted to investigate the relevance of non-additive genetic effects on sire conception rate, the US national phenotypic evaluation of dairy bull fertility.

### Significant non-additive effects

**Fig. 2:**
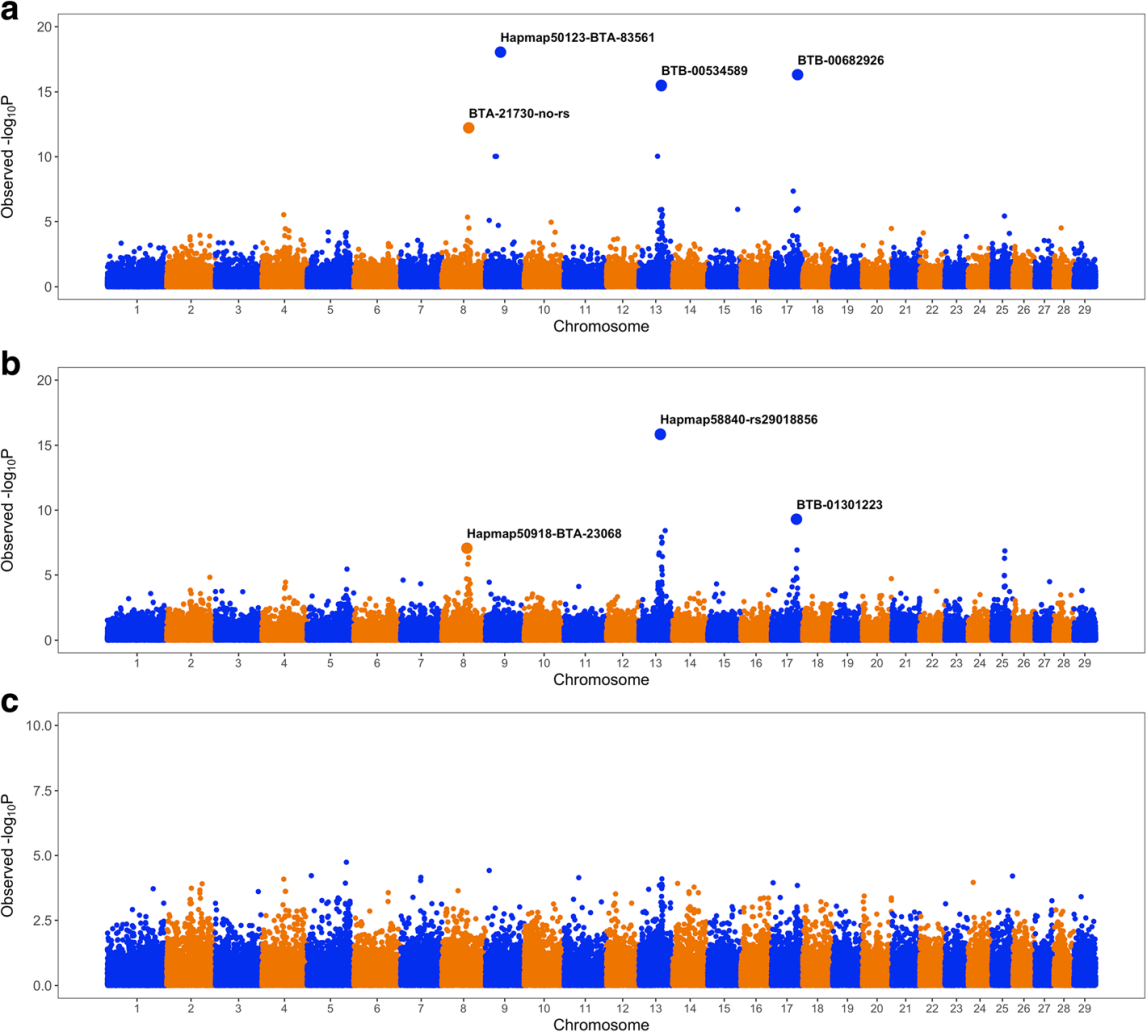
Manhattan plots showing the significance of dominance (**a**), recessive (**b**), and overdominance (**c**) effects on Sire Conception Rate across the entire bovine genome

**Fig. 3:**
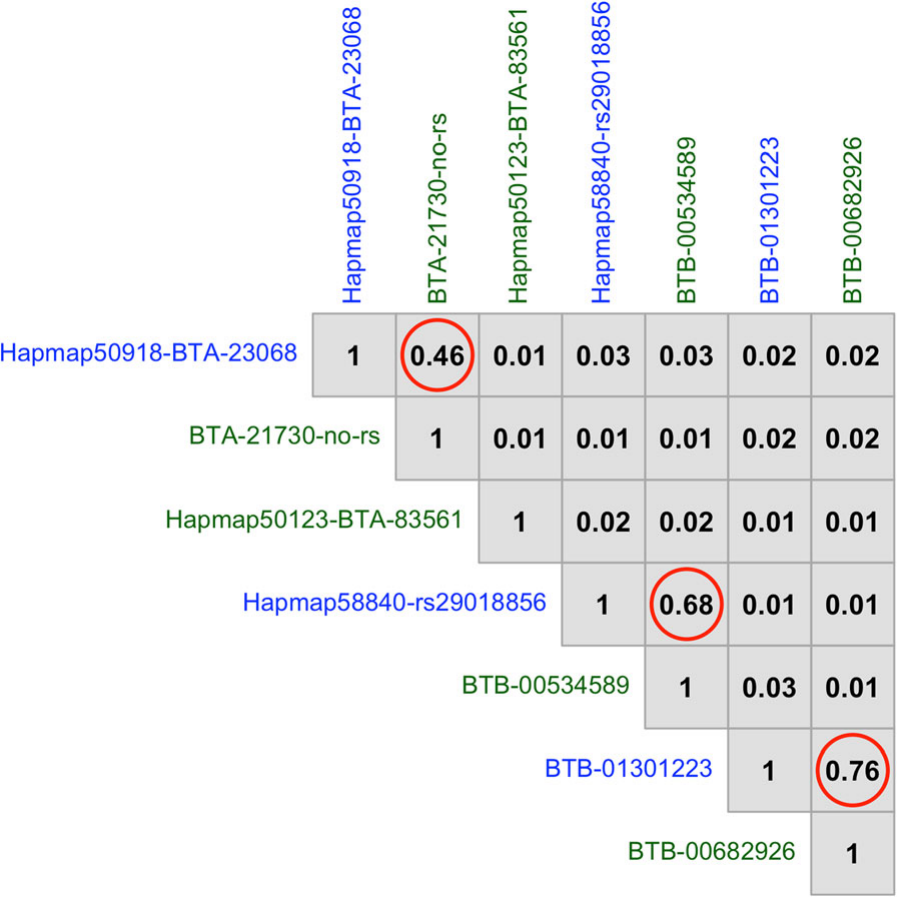
Pattern of linkage disequilibrium (*r*^2^) among the most significant non-additive SNP markers. Dominance and recessive markers are shown in green and blue, respectively. Markers *Hapmap50918-BTA-23068* and *BTA-21730-no-rs* are located on BTA8; *Hapmap50123-BTA-83561* is located on BTA9; *Hapmap58840-rs29018856* and *BTB-00534589* are located on BTA13; *BTB-01301223* and *BTB-00682926* are located on BTA17

Figure 2 shows the results of the whole-genome single-marker scans for testing dominance, recessive, and overdominance effects on SCR. Four different genomic regions, distributed on chromosomes BTA8, BTA9, BTA13, and BTA17, showed either significant dominance or recessive effects after Bonferroni correction (adjusted *Pvalue* < 0.01). Table 1 describes in detail the most significant SNP loci, including position, allelic and genotypic frequencies, and genetic effects. Importantly, these loci showed negligible (non-significant) additive effects, and hence, we can infer that the mode of gene action of these markers is purely non-additive. The pattern of linkage disequilibrium among these markers is shown in Fig. 3. As expected, markers in different chromosomes were in linkage equilibrium. However, markers in the same genomic regions were highly correlated (high linkage disequilibrium), and hence, it is very likely that they represent the same genetic signal. Regarding overdominance effects, no significant marks were detected after correction for multiple testing (Fig. 2c).

**Table 1:** Most significant non-additive genetic markers associated with Sire Conception Rate

Marker	Chr.	Position	MAF	Gen Freq.	β ± se	*P*_*NonAdd*_	*P*_*Add*_
Dominance							
BTA-21730-no-rs	8	74,336,311	0.145	145 (AA)	1.33 ± 0.18	5.9 × 10^−13^	1.1 × 10^−05^
				1870 (AB)			
				5443 (BB)			
Hapmap50123-BTA-83561	9	43,054,017	0.062	17 (AA)	4.49 ± 0.51	9.2 × 10^−19^	3.1 × 10^−02^
				885 (AB)			
				6556 (BB)			
BTB-00534589	13	60,300,299	0.150	148 (AA)	1.47 ± 0.18	3.3 × 10^−16^	1.4 × 10^−03^
				1942 (AB)			
				5367 (BB)			
BTB-00682926	17	66,268,354	0.142	103 (AA)	1.79 ± 0.21	5.0 × 10^−17^	1.6 × 10^−02^
				1917 (AB)			
				5437 (BB)			
Recessive							
Hapmap50918-BTA-23068	8	68,404,576	0.256	4095 (AA)	−0.60 ± 0.11	8.6 × 10^−08^	3.5 × 10^−02^
				2905 (AB)			
				458 (BB)			
Hapmap58840-rs29018856	13	58,529,087	0.128	5644 (AA)	−1.81 ± 0.22	1.5 × 10^−16^	2.7 × 10^−03^
				1715 (AB)			
				99 (BB)			
BTB-01301223	17	64,049,290	0.171	5073 (AA)	−1.06 ± 0.17	5.0 × 10^−10^	5.6 × 10^−02^
				2219 (AB)			
				166 (BB)			

*MAF* minor allele frequency

**Fig. 4:**
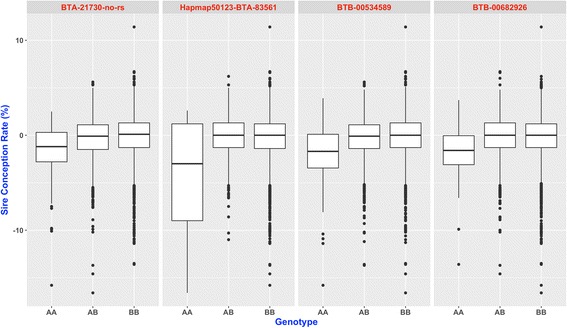
Box plot showing the distribution of Sire Conception Rate phenotypes for four SNP loci with marked dominance effects

The distribution of SCR values for each of the four SNP loci with significant dominance effects is displayed in Fig. 4. These box plots clearly show that AB and BB genotypes have higher SCR values than AA genotypes. Notably, the differences between SCR means are roughly 1.5 and 2 points, depending on the locus. In other words, each of these loci is explaining observed differences in conception rates between AB/BB and AA bulls of almost 2%.

### Candidate genes in significant regions

The significant region in BTA8 located at 73–76 Mb harbors at least two candidate genes, namely *ADAM28* and *DNAJA1*, that might be directly related to service sire fertility. Gene *ADAM28* encodes a member of the disintegrin and metalloproteinase family, a group of membrane proteins implicated in a variety of biological processes involving cell-cell and cell-matrix interactions, including gametogenesis and fertilization [28]. In particular, *ADAM28* is predominantly expressed in the epididymis, denoting a direct role in sperm maturation [29]. Moreover, gene *DNAJA1* is a member of the DnaJ family, a group of molecular chaperons that assists other proteins in their folding, transport and assembly into complexes [30]. Interestingly, it has been shown that *DNAJA1* plays an important role in testis development and spermatogenesis through the regulation of androgen receptor signaling in Sertoli cells [31]. Indeed, the loss of *DNAJA1* function in mice results in the accumulation of androgen receptor protein in Sertoli cells, which in turn hyperactivates the androgen receptor signaling pathway, leading to testicular hypogonadism and severe defects in spermatogenesis [31].

At least four putative candidate genes for bull fertility, *TBC1D20*, *SPO11*, *RAD21L1* and *BCL2L1*, are located in the significant region detected in BTA13 at 58–62 Mb. Gene *TBC1D20* encodes a GTPase-activating protein that is directly implicated in sperm biology, especially in acrosome assembly [32]. Indeed, the disruption of *TBC1D20* gene in mice results in defects in the acrosomal formation, which in turn leads to male infertility [33]. Moreover, two of the candidate genes, *SPO11* and *RAD21L1* are directly implicated in the meiosis, a key step during gametogenesis. Gene *SPO11* is a topoisomerase-like protein responsible for the formation of DNA double-strand breaks that occur during meiotic recombination [34]. It has been shown in mice that the disruption of *SPO11* leads to severe gonadal abnormalities, including the apoptotic death of spermatocytes during early meiosis [35]. Interestingly, a recently case-control study in humans identified a SNP in exon 7 of *SPO11* significantly associated with male infertility [36]. On the other hand, *RAD21L1* encodes a protein that is part of large multi-subunit complexes, known as cohesins, that play important roles in many aspects of meiotic chromosome dynamics [37]. Recent evidence suggests that *RAD21L1* is specially implicated in male meiosis; indeed, the disruption of *RAD21L* in mice precludes the initial association of homologous chromosome as well as the subsequent pairing in spermatocytes [38]. Finally, *BCL2L1* encodes a member of the BCL-2 protein family, a well-studied group of proteins that either promote or inhibit apoptosis [39]. Notably, *BCL2L1* is directly involved in the regulation of germ line apoptosis in the testis [40].

The region in BTA17 located at 64–68 Mb harbors two putative genes for service sire fertility, *PIWIL3* and *TMEM119*. Gene *PIWIL3* encodes a member of the PIWI family, a group of proteins that appear to be restricted to the germ line and bind to a subclass of germ cell-specific small RNAs [41]. These proteins are essential for spermatogenesis controlling post-transcriptional events and subsequent translation, and exerting control over transposons [41]. Notably, a non-synonymous SNP in *PIWIL3* was significantly associated with an altered risk of oligozoospermia in humans [42]. Moreover, *TMEM119* encodes a transmembrane protein actively involved in osteoblast differentiation. Recent studies have shown that *TMEM119* also affects different male fertility traits. The gene *TMEM119* is expressed in spermatocytes and spermatids in the developing testis, and *TMEM119*-deficient mice showed impaired testis development and spermatogenesis, resulting in a significant decrease in both testis weight and sperm number [43]. Current evidence suggests that the absence of *TMEM119* perturbs late spermatogenesis, specifically at the round spermatid stage [43].

## Conclusions

In this study, we investigated the relevance of non-additive effects on bull fertility in Holstein cattle. Four genomic regions in BTA8, BTA9, BTA13, and BTA17 showed marked dominance and recessive effects. These regions harbors genes with known roles in male fertility, including testis development, spermatogenesis, and sperm maturation. Overall, our results provide more evidence for the relevance of non-additive effects in traits closely related to fitness such as male fertility. In addition, these findings may contribute to the development of new strategies for improving sire fertility via selective breeding and marker-assisted selection.

## Abbreviations

*SCR:* Sire Conception Rate: *SNP:* Single Nucleotide Polymorphism
